# Targeted generation of polyploids in *Hydrangea macrophylla* through cross-based breeding

**DOI:** 10.1186/s12863-020-00954-z

**Published:** 2020-12-07

**Authors:** Conny Tränkner, Kristina Günther, Peter Sahr, Frauke Engel, Annette Hohe

**Affiliations:** 1 Leibniz Institute of Vegetable and Ornamental Crops, Theodor-Echtermeyer-Weg 1, 14979 Großbeeren, Germany; 2 Present address: Erfurt Research Centre for Horticultural Crops, University of Applied Sciences Erfurt, Kühnhäuser Straße 101, 99090 Erfurt, Germany; 3 Present address: Julius Kühn-Institut (JKI) Federal Research Centre for Cultivated Plants, Institute for Breeding Research on Agricultural Crops, Groß Lüsewitz, Rudolf-Schick-Platz 3a, 18190 Sanitz, Germany; 4 Gartenbau Kötterheinrich-Hortensienkulturen, Hohner Mark 20, 49525 Lengerich, Germany; 5 Faculty of Landscape Architecture, Horticulture and Forestry, University of Applied Sciences Erfurt, Leipziger Straße 77, 99085 Erfurt, Germany

**Keywords:** Aneuploidy, Chromosome number, DNA content, Flow cytometry, Ornamental, Polyploidy, Unreduced gametes

## Abstract

**Background:**

Up to now, diploid and triploid cultivars were reported for the ornamental crop *Hydrangea macrophylla*. Especially, the origin of triploids and their crossing behaviors are unknown, but the underlying mechanisms are highly relevant for breeding polyploids.

**Results:**

By screening a cultivar collection, we identified diploid, triploid, tetraploid and even aneuploid *H. macrophylla* varieties. The pollen viability of triploids and tetraploids was comparable to that of diploids. Systematic crosses with these cultivars resulted in viable diploid, triploid, tetraploid and aneuploid offspring. Interestingly, crosses between diploids produced diploid and 0 or 1–94% triploid offspring, depending on the cultivars used as pollen parent. This finding suggests that specific diploids form unreduced pollen, either at low or high frequencies. In contrast, crosses of triploids with diploids or tetraploids produced many viable aneuploids, whose 2C DNA contents ranged between the parental 2C values. As expected, crosses between diploid and tetraploid individuals generated triploid offspring. Putative tetraploid plants were obtained at low frequencies in crosses between diploids and in interploid crosses of triploids with either diploid or tetraploid plants. The analysis of offspring populations indicated the production of *1n* = *2x* gametes for tetraploid plants, whereas triploids produced obviously reduced, aneuploid gametes with chromosome numbers ranging between haploid and diploid level. While euploid offspring grew normally, aneuploid plants showed mostly an abnormal development and a huge phenotypic variation within offspring populations, most likely due to the variation in chromosome numbers. Subsequent crosses with putative diploid, triploid and aneuploid offspring plants from interploid crosses resulted in viable offspring and germination rates ranging from 21 to 100%.

**Conclusions:**

The existence of diploids that form unreduced pollen and of tetraploids allows the targeted breeding of polyploid *H. macrophylla*. Different ploidy levels can be addressed by combining the appropriate crossing partners. In contrast to artificial polyploidization, cross-based polyploidization is easy, cheap and results in genetically variable offspring that allows the direct selection of more robust and stress tolerant polyploid varieties. Furthermore, the generation of polyploid *H. macrophylla* plants will favor interspecific breeding programs within the genus *Hydrangea*.

**Supplementary Information:**

The online version contains supplementary material available at 10.1186/s12863-020-00954-z.

## Background

Polyploid organisms contain more than two complete sets of chromosomes per cell nucleus. This often results in an increased fitness, higher yield, improved product quality and better tolerance to abiotic and biotic stresses in comparison with diploid relatives. Moreover, polyploid plants may produce larger and more beautiful flowers, tend to flower later or show elongated flowering periods compared to related diploids [1]. Thus, the production of polyploid plants is an important strategy of crop breeding, especially in ornamental crops.

*Hydrangea macrophylla* (Thunb.) Ser. is an economically important ornamental crop, which belongs to the upmarket segment. This perennial, deciduous species produces attractive foliage and large, colorful inflorescences. Its cultivars are used for indoor and outdoor cultivation, for landscaping and for production of fresh and dried flowers for florists. Important traits in breeding of *H. macrophylla* are brightly colored flowers arranged in impressive inflorescences, attractive foliage, remontant flowering as well as compact growth. Further relevant traits are tolerance against biotic and abiotic stress and various characteristics that are important for efficient production. *H. macrophylla* is an obligate out-crossing species [2–4], and therefore highly heterozygous. Heterozygosity of the parental plants and genetic variability between the different crossing partners result in genotypic and phenotypic variation already in the F_1_ generation. Thus, F_1_ plants can be used for selection. Subsequent plant propagation occurs vegetatively through cuttings.

The existence of diploid and triploid cultivars in *H. macrophylla* has first been reported by Demilly et al. [5]. Diploid plants of *H. macrophylla* contain *2n* = *2x* = 36 chromosomes and show 2C DNA contents from 3.9 to 5.0 pg. In contrast, triploid plants have *2n* = *3x* = 54 chromosomes, and 2C DNA contents vary between 6.5 and 7.3 pg [6–11]. Recently, a cultivar has been found with a 2C DNA content of 8.9 pg, suggesting tetraploidy [12]. About 30 triploid cultivars were reported until now [7–10]. Some of these cultivars date back to a breeding program that has been undertaken in a research station in Wädenswil (Switzerland) in the middle of the twentieth century [13]. Based on flow cytometric and genotypic data, the Wädenswil pedigree of 26 *H. macrophylla* lacecap cultivars has been reconstructed [9]. This reconstruction showed that triploid cultivars were produced independently (and most likely unwittingly) in different crosses. Furthermore, this reconstruction revealed crosses between diploid and triploid plants that obviously resulted in viable offspring [9].

The breeding of polyploid *H. macrophylla* is of high interest. Artificial polyploidization has been successfully demonstrated for the diploid *H. macrophylla* cultivars ‘Adria’ and ‘Libelle’ and the triploid cultivars ‘Blaumeise’ and ‘Nachtigall’. It resulted in tetraploid and hexaploid plants. While the tetraploid plants developed normally and were attractive as the diploid initial cultivars, the hexaploid plants showed growth depression, leaf deformation and hampered flower development [7]. Although artificial polyploidization was successfully applied in *H. macrophylla*, it is labor and cost intensive. Furthermore, it does not explain the high frequency of existing triploid cultivars of *H. macrophylla*.

Jones et al. [8] hypothesized that triploid *H. macrophylla* cultivars were obtained unwittingly by crossing diploids with a coincidentally existing tetraploid individual or by using triploids in crosses. The latter is possible, because triploid individuals of *H. macrophylla* are at least partially fertile in crosses when used as seed or pollen parent [8]. Spontaneous polyploidization was observed when the diploid *H. macrophylla* cultivars ‘Princess Juliana’ and ‘Trophee’ were crossed. This cross combination resulted in 94% triploids when ‘Trophee’ was used as pollen parent, whereas only diploids were produced in the reciprocal cross [11]. Due to the bimodal pollen size of ‘Trophee’, the author suggested that unreduced pollen might be causative for the production of these triploids.

The main process of triploid production is widely unknown, particularly with regard to interploid crosses between diploid and triploid plants of *H. macrophylla*. Furthermore, it is unknown whether triploids produce offspring populations that are suitable for cultivar selection. However, the knowledge about both of these issues will support the targeted breeding of triploid and even tetraploid *H. macrophylla*.

In this study, we realized extensive and systematic crosses between diploid, triploid and tetraploid individuals of *H. macrophylla* and analyzed the different F_1_ populations by flow cytometry. In this way, we aimed to reveal

i) to which extent crosses between cultivars of different ploidy levels are successful,
ii) whether offspring from interploid crosses is viable and suitable for selection and further crossings,
iii) which cross combinations allow the targeted generation of polyploids,
iv) which ploidy (or aneuploidy) is existent in gametes produced by diploid, triploid and tetraploid crossing partners.

Based on our findings, we developed a systematic breeding strategy for the targeted generation of polyploids in *H. macrophylla*.

## Results

### Interploid crosses generate viable offspring

Previously, we had characterized a *H. macrophylla* collection comprising 80 varieties. Through flow cytometric analysis, we had identified putative diploid, triploid and tetraploid cultivars, which showed 2C DNA contents between 3.95 and 4.61 pg for diploid, 6.47 and 6.97 pg for triploid and 8.85 pg for tetraploid plants [9, 12]. In the present study, we determined the ploidy of 8 putative diploid, 6 triploid and 1 tetraploid cultivar(s) by chromosome counting. We identified 7 diploid, 6 triploid and one tetraploid genotype(s) containing *2n* = *2x* = 36, *2n* = *3x* = 54 and *2n* = *4x* = 72 chromosomes, respectively (Table 1, Figure S1). Surprisingly, the cultivar ‘Mücke II’ [9] had *2n* = *2x* + 1 = 37 chromosomes at a 2C DNA content of 4.45 pg and was determined as aneuploid instead of diploid (Figure S1).

**Table 1:** Characterization of *H. macrophylla* cultivars used for intraploid and interploid crosses

	Cultivar	SSR fingerprint ID^a^	n^b^	2C DNA content (mean ± SD) [pg]	Counted chromosomes	Pollen viability (mean ± SD) [%]
Diploid (*n* = 12)	Baby Blue	G75	7	4.49 ± 0.01	36	55 ± 7
	Bläuling	G07	6	4.46 ± 0.02	36	59 ± 5
	Bodensee^c^	G09	5	4.51 ± 0.03	36	49 ± 6
	Choco Bleu	G68	6	4.49 ± 0.02	36	54 ± 5
	Dark Angel	G72	4	4.35 ± 0.04	36	26 ± 5
	Hörnli	G30	3	4.47 ± 0.01	36	56 ± 5
	Forever Pink	G73	1	4.61	36	n.d.
	Libelle	G33	9	4.45 ± 0.02	36	33 ± 2
	Little Prince	G69	7	4.44 ± 0.01	36	36 ± 7
	Paris	G70	5	4.40 ± 0.03	36	n.d.
	Sheila	G71	3	4.43 ± 0.03	36	63 ± 6
	Sweet Dreams	G78	2	4.40 ± 0.03	36	61 ± 5
Triploid (*n* = 6)	Bela	G02	8	6.60 ± 0.04	54	27 ± 6
	Blaumeise	G02	6	6.49 ± 0.02	54	38 ± 6
	Enziandom^c^	G17	7	6.71 ± 0.04	54	26 ± 6
	Oregon Pride	n.d.	3	6.64 ± 0.05	54	17 ± 1
	R.F. Felton	G79	4	6.89 ± 0.06	54	58 ± 8
	Zorro	n.d.	8	6.59 ± 0.03	54	51 ± 3
Tetraploid (*n* = 1)	Benelux	G03	3	9.04 ± 0.03	72	61 ± 8

^a^according to Hempel et al. [9] and Tränkner et al. [12], ^b^number of samples measured by flow cytometry, ^c^several different genotypes were found under this cultivar name according to Hempel et al. [9], n.d. not determined

In order to reveal crossing strategies to generate polyploid *H. macrophylla* plants sexually, we selected 12 diploid, 6 triploid and 1 tetraploid *H. macrophylla* cultivar(s) for crossing experiments (Table 1). The pollen viability of the parental plants ranged from 26 to 63% (mean 49 ± 13%) for diploids, from 17 to 58% (mean 36 ± 16%) for triploids and was 61% for the tetraploid cultivar (Table 1). Although pollen viability was more frequently reduced in triploid cultivars, significant differences between diploids and triploids were not observed (Student’s t test, *p* = 0.096, α = 0.05). In total, all plants produced sufficient amounts of viable pollen and were suitable for subsequent crosses.

In total, we studied 44 cross combinations and 6 selfings. Each combination was represented by 1 to 7 hand crosses (120 crosses in total), which were performed in the years from 2013 to 2017. All crosses and selfings are summarized in Table 2. As expected, all selfings failed to produce seeds, which reflects the widespread self-incompatibility of *H. macrophylla*. In contrast, 25 out of 44 cross combinations between different cultivars succeeded, 8 were difficult and 11 combinations failed. Nine intraploid cross combinations between 6 different diploid cultivars were included as a control. Eight out of these 9 crosses (89%) were successful. In interploid crosses between diploids and triploids, 12 out of 16 cross combinations (75%) were successful irrespective of the cross direction. Crosses between diploid and tetraploid and between triploid and tetraploid plants were successful in 3 out of 7 (43%) and in 2 out of 4 (50%) combinations, respectively. In contrast, all 8 intraploid crosses between different triploids failed: 6 combinations yielded no seeds, and 2 combinations resulted in only 3 and 10 seeds (Table 2).

**Table 2:** Summary of crosses. Crosses with more than 20 seeds were defined as successful, with 1–20 seeds as difficult and without seeds as not successful

Cross type	Seed bearer	Pollinizer	Number of crosses	Crossing success	Germination rate [%]	Number of F_1_ plants analyzed by flow cytometry	Description of the F_1_ population regarding ploidy
selfings (*n* = 6)	Baby Blue		1	not successful	–	–	–
	Bela		3	not successful	–	–	–
	Benelux		1	not successful	–	–	–
	Enziandom		2	not successful	–	–	–
	Hörnli		1	not successful	–	–	–
	R.F. Felton		1	not successful	–	–	–
diploid x diploid (*n* = 9)	Dark Angel	Paris	3	successful	76	60	58 diploids, 2 triploids
	Paris	Dark Angel	1	successful	n.d.	424^a^	105 diploids, 317 triploids, 2 aneuploids
	Dark Angel	Sheila	4	successful	52	54	54 diploids
	Sheila	Dark Angel	4	successful	47	68	34 diploids, 34 triploids
	Dark Angel	Hörnli	4	not successful	–	–	–
	Hörnli	Dark Angel	4	successful	81	60	19 diploids, 40 triploids, 1 tetraploid
	Hörnli	Sheila	3	successful	45	60	60 diploids
	Sheila	Hörnli	5	successful	64	34	2 diploids, 31 triploids, 1 tetraploid
	Sweet Dreams	Bläuling	7	successful	46	70^a^	69 diploids, 1 triploid
diploid x triploid (*n* = 7)	Baby Blue	Bela	1	difficult	n.d.	4	aneuploids between 2x and 3x level
	Baby Blue	Enziandom	2	difficult	n.d.	1	aneuploid between 2x and 3x level
	Bodensee	Bela	4	successful	60	61	aneuploids between 2x and 3x level
	Choco Bleu	Zorro	4	successful	32	26	aneuploids between 2x and 3x level
	Libelle	Enziandom	5	successful	59	76	aneuploids between 2x and 3x level
	Little Prince	Enziandom	4	successful	41	35	aneuploids between 2x and 3x level
	Sweet Dreams	Blaumeise	1	successful	64	14^a^	aneuploids between 2x and 4x level
triploid x diploid (*n* = 9)	Bela	Baby Blue	1	not successful	–	–	–
	Bela	Bodensee	5	successful	42	63	aneuploids between 2x and 3x level
	Blaumeise	Sweet Dreams	1	successful	42	24^a^	aneuploids between 2x and 4x level
	Enziandom	Baby Blue	1	successful	n.d.	37	aneuploids between 2x and 3x level
	Enziandom	Libelle	3	successful	28	35	aneuploids between 2x and 3x level
	Enziandom	Little Prince	3	successful	44	42	aneuploids between 2x and 3x level
	Oregon Pride	Choco Bleu	1	difficult	n.d.	3	aneuploids between 2x and 3x level
	R.F. Felton	Baby Blue	2	successful	47	59	aneuploids between 2x and 3x level
	Zorro	Choco Bleu	5	successful	82	110	aneuploids between 2x and 4x level
triploid x triploid (*n* = 8)	Bela	Enziandom	4	not successful	–	–	–
	Enziandom	Bela	3	difficult	n.d.	3	n.d.
	Bela	R.F. Felton	1	not successful	–	–	–
	R.F. Felton	Bela	1	not successful	–	–	–
	Enziandom	R.F. Felton	1	not successful	–	–	–
	R.F. Felton	Enziandom	1	not successful	–	–	–
	Enziandom	Zorro	4	difficult	n.d.	10	aneuploids between 2x and 4x level
	Zorro	Enziandom	3	not successful	–	–	–
diploid x tetraploid (*n* = 3)	Baby Blue	Benelux	1	not successful	–	–	–
	Bodensee	Benelux	4	successful	46	83	triploids
	Little Prince	Benelux	1	not successful	–	–	–
tetraploid x diploid (*n* = 4)	Benelux	Baby Blue	1	not successful	–	–	–
	Benelux	Bodensee	5	successful	54	60	triploids
	Benelux	Forever Pink	1	successful	n.d.	58	triploids
	Benelux	Little Prince	1	difficult	n.d.	1	triploid
triploid x tetraploid (*n* = 2)	Bela	Benelux	4	successful	34	192	aneuploids between 3x and 4x level
	Enziandom	Benelux	3	difficult	n.d.	4	aneuploids between 2x and 4x level
tetraploid x triploid (*n* = 2)	Benelux	Bela	1	successful	n.d.	136	aneuploids between 3x and 4x level
	Benelux	Enziandom	2	difficult	n.d.	1	aneuploid between 3x and 4x level

^a^Data presented already in Tränkner et al. [12, 14], n.d. not determined

Subsequently, we determined the germination rates of seeds from 21 successful cross combinations. The results are shown in Table 2. The germination rates of 7 intraploid crosses between diploids ranged from 45 to 81% (mean 59 ± 16%). For 11 interploid crosses between diploid and triploid plants, we determined germination rates from 28 to 82% (mean 49 ± 16%). Two crosses between diploid and tetraploid plants showed germination rates of 46 and 54%, while 34% germinating seeds were observed for a cross between a triploid and a tetraploid plant. The germination rates of intraploid and interploid crosses showed no significant differences (Student’s t test, *p* = 0.137, α = 0.05). Hence, nearly all diploid, triploid and tetraploid cultivars used in these crosses were fertile. Interploid crosses between diploid, triploid and tetraploid plants were mostly successful and resulted in viable offspring. Thus, triploidy and tetraploidy caused *per se* no crossing barriers in *H. macrophylla* and triploid as well as tetraploid *H. macrophylla* cultivars can be used for crossings. However, general crossing barriers seem to exist for crosses between triploids.

### Crosses between diploids result in diploid, triploid and tetraploid progenies

**Fig. 1:**
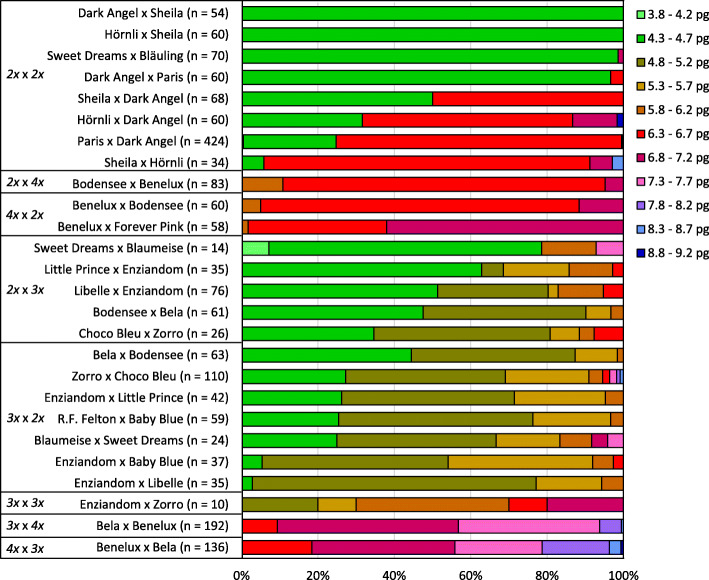
Distribution of 2C DNA contents of F_1_ plants from intraploid and interploid crosses

**Fig. 2:**
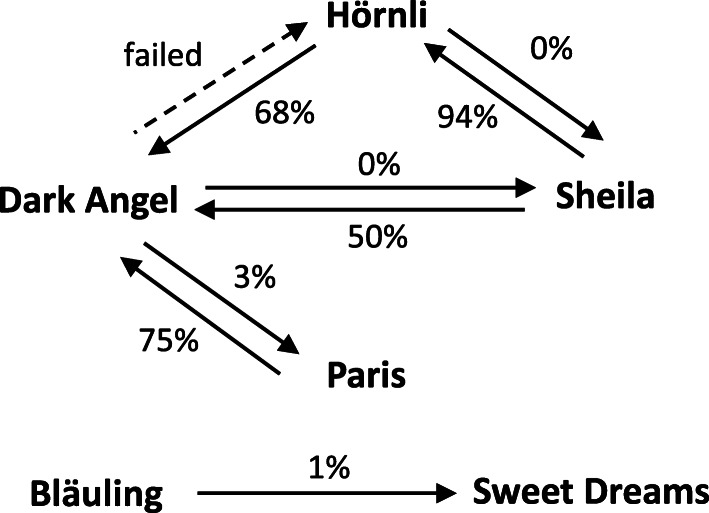
Crosses between diploid cultivars result in diploid and polyploid offspring plants. The proportion of polyploid progenies is given in %. In reciprocal crosses, the frequencies of spontaneously polyploidized progenies depend on the cross direction. The arrow points to the cultivar that was used as pollinizer

In order to study the ploidy level in plants of the next generation, we determined the 2C DNA content of F_1_ plants. First, we analyzed 8 F_1_ populations derived from intraploid crosses between diploids. These populations included 34 to 424 plants. The 2C values are summarized per population in Fig. 1 and presented for each plant per population in Figure S2. Two of these populations produced offspring plants with 2C DNA contents between 4.36 and 4.56 pg, on average 4.44 ± 0.04 pg (*n* = 54) and 4.48 ± 0.03 pg (*n* = 60), respectively. These 2C values indicated diploidy for all of these F_1_ plants. In contrast, the other 6 diploid-diploid populations generated diploid plants as well as 1 to 319 plants with 2C DNA contents more than 6.0 pg, clearly exceeding the 2C DNA content of a diploid *H. macrophylla* plant. In these populations, the 2C DNA contents grouped into three discrete intervals: 4.10 to 4.67 pg (mean 4.44 ± 0.08 pg, *n* = 285), 6.35 to 6.89 pg (mean 6.57 ± 0.08 pg, *n* = 425) and 8.45 to 8.88 pg (mean 8.67 ± 0.22 pg, *n* = 3), indicating diploidy, triploidy and tetraploidy. As shown in Table 2, 2 out of these 6 populations included 1 to 2 putative triploid F_1_ plants (1.4 and 3.3%), whereas 4 populations contained 32 to 317 putative triploid (50.0 to 91.2%) and sporadically even tetraploid F_1_ plants (Figure S2). While 1 to 2 triploid and tetraploid F_1_ plants might have arisen randomly, a fixed mechanism seems to be responsible for the development of high proportions of polyploid plants. These high rates of polyploid offspring were observed only when ‘Dark Angel’ or ‘Hörnli’ were used as pollen parents. In contrast, when these cultivars were used as seed parents, they produced diploid and 0 to 3.3% triploid offspring (Fig. 2, Figure S2). Hence, it seems that some *H. macrophylla* cultivars form partially unreduced gametes, primarily unreduced pollen, and that unreduced pollen is produced genotype-specifically either at low or high frequencies.

**Fig. 3:**
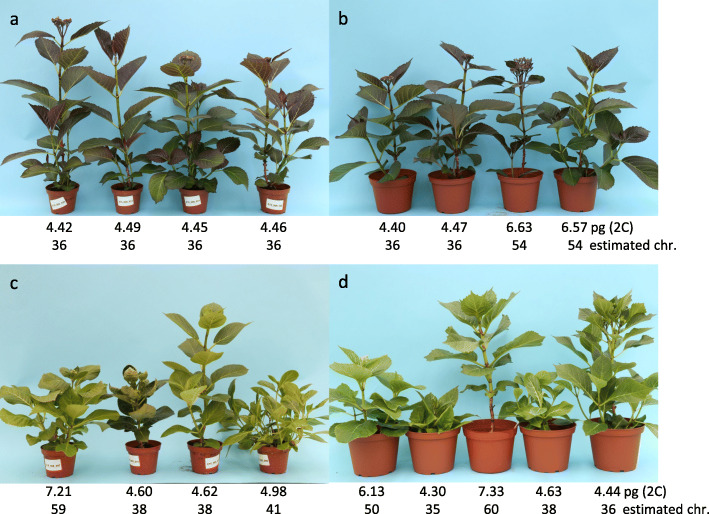
Phenotype, 2C DNA content and estimated chromosome numbers of progenies from crosses between diploids (top) and interploid crosses between diploid and triploid plants (bottom). The cross ‘Dark Angel’ x ‘Sheila’ produced diploid offspring (**a**), whereas the reciprocal cross ‘Sheila’ x ‘Dark Angel’ resulted in diploid and triploid progenies (**b**), all with normal development. The cross ‘Blaumeise’ x ‘Sweet Dreams’ (**c**) and the reciprocal cross ‘Sweet Dreams’ x ‘Blaumeise’ (**d**) resulted in aneuploid offspring between diploid and tetraploid level with abnormal phenotypes

All progenies from crosses between diploids showed a normal development, independently of the ploidy level. Within populations, plants looked similar and differed less than between populations. A differentiation between diploid, triploid and tetraploid progenies was not possible by eye (Fig. 3a, b).

### Crosses between diploids and tetraploids generate triploid offspring

In a wide range of plant species, triploid plants can be generated by crossing diploid with tetraploid individuals. Correspondingly, we studied the offspring of 4 cross combinations between 3 diploid *H. macrophylla* cultivars and the tetraploid cultivar ‘Benelux’. Each population included 1 to 83 F_1_ plants (Table 2). In total, 202 F_1_ plants were analyzed by flow cytometry. The 2C DNA contents of these plants ranged from 6.08 to 7.18 pg, on average 6.59 ± 0.20 pg (Fig. 1, Figure S2). The majority of these plants had 2C values within the range of triploid *H. macrophylla* cultivars [9, 12], and we suggested triploidy for all of these F_1_ plants. Hence, crosses between tetraploid and diploid *H. macrophylla* individuals produced exclusively triploid plants as expected. Our results show that the tetraploid cultivar ‘Benelux’ performs a regular meiosis and produces maternally and paternally *1n* = *2x* gametes. Plant phenotyping was not done for these populations.

### Crossing triploids with diploids or tetraploids result in predominantly aneuploid offspring

Triploid plants are often considered to be sterile due to disturbed meiosis. However, 75% of cross combinations between triploid and diploid *H. macrophylla* cultivars were successful. Subsequently, we studied the 2C DNA content of 217 and 373 F_1_ plants, respectively, derived from 7 diploid x triploid and 8 triploid x diploid crosses. The 2C DNA contents of these F_1_ plants ranged from 3.87 to 8.50 pg, on average 5.02 ± 0.55 pg. As summarized in Fig. 1 and shown for each plant per population in Figure S2, all combinations produced aneuploid progenies with differing 2C DNA contents. These 2C DNA contents ranged between the 2C values of the corresponding diploid and triploid parents. When the triploid cultivar was used as pollen parent, 2C DNA contents between 4.3 and 5.2 pg predominated. When the triploids were used as seed parent, less F_1_ plants with 2C values between 4.3 and 4.7 pg and more F_1_ plants with 2C values between 4.8 and 5.7 pg were generated (Fig. 1), suggesting an effect of the cross direction. Furthermore, 3 combinations generated also F_1_ plants with 2C DNA contents above the triploid level (Fig. 1, Figure S2).

Hence, triploids seem to perform an unbalanced separation of homologous chromosomes during meiosis, which obviously generates male as well as female gametes with varying numbers of chromosomes. 2C DNA contents above triploid level might be caused through partially produced, unreduced gametes or through gametes, which have enriched 2 and 3 homologous chromosomes by unbalanced separation. In contrast, 2C DNA contents below diploid level might be caused through gametes, which lack some chromosomes completely.

Although intraploid crosses between different triploids were generally not successful, we had obtained in total 10 F_1_ plants from 4 hand crosses between ‘Enziandom’ and ‘Zorro’. The 2C DNA content of these plants ranged from 5.15 to 7.03 pg (Fig. 1, Figure S2). Most plants showed 2C values below triploid level, suggesting that gametes with lower DNA contents might have a selective advantage in generating viable offspring. As mentioned above, triploid *H. macrophylla* individuals produce female as well as male gametes with varying numbers of chromosomes. Combinations of aneuploid gametes seem to result in even stronger genetic disturbance, which might explain the crossing barrier between triploid *H. macrophylla* plants.

Four different cross combinations between triploid and tetraploid plants resulted in total in 333 F_1_ plants (Table 2). The 2C DNA contents of these plants ranged from 5.51 to 9.05 pg, on average 7.22 ± 0.44 pg. Most of these F_1_ plants showed 2C values between the triploid and tetraploid level indicating aneuploidy in the range between 54 and 72 chromosomes (Fig. 1, in detail Figure S2). In accordance with the results observed for crosses between diploid and triploid plants, we suggest that aneuploidy is caused by the triploid parent due to gametes with varying numbers of chromosomes.

Most of the generated F_1_ plants showed an abnormal growth, irrespective of whether the triploid plant was used as seed or pollen parent (Fig. 3c, d). Plants were stunted or showed abnormal elongated growth. The size, form, serration and color of leaves differed strongly between offspring plants. Some plants did not produce flowers, whereas other plants produced flowers with partially deformed floral organs. The phenotypic variation within these populations was clearly stronger compared to the F_1_ populations derived from crosses between diploids or between diploids and tetraploids.

### Interploid crosses generate fertile offspring

In order to test the fertility of offspring derived from interploid crosses, we performed test crosses of putative diploid, triploid and aneuploid F_1_ plants with diploid *H. macrophylla* cultivars. For this purpose, we selected 23 F_1_ plants from 5 interploid cross combinations between diploid, triploid and tetraploid *H. macrophylla* cultivars (Table S1). The 2C DNA content of these F_1_ plants ranged from 4.38 to 7.72 pg. Sixteen out of these 23 F_1_ plants produced pollen, whose viability ranged from 4 to 43%, whereas 6 F_1_ plants produced no pollen. All F_1_ plants were used as seed parent. In case they produced pollen, also reciprocal crosses were performed. The cultivars ‘Bläuling’, ‘Libelle’, ‘Baby Blue’ and ‘Sheila’ were chosen as crossing partners. These cultivars are diploid and were successfully used in previous crosses. In total, we performed 62 crosses. As shown in Table S1**,** 31 (50%) crosses were successful, 14 difficult and 17 crosses failed. Crosses with plants derived from diploid-triploid and triploid-tetraploid crosses were at least partially successful, irrespective of whether or not the F_1_ plants were euploid and independent of the cross direction. Neither variation in ancestry, 2C DNA content nor pollen viability could account for the success or failure of these crosses. Subsequently, we sowed between 24 and 250 seeds per successful cross. Each of these sowings produced viable seedlings. We determined germination rates ranging from 20.8 to 100.0% (Table S1). Although we did not phenotype theses progenies further, our results show that offspring plants from interploid crosses can be used successfully in further crossings.

## Discussion

Until now, only the existence of diploid and triploid *H. macrophylla* cultivars was reported [5, 8–10]. The origin of these triploid *H. macrophylla* cultivars was unknown. The two general mechanisms for polyploidization are autopolyploidy and allopolyploidy. Autopolyploidy occurs through somatic doubling or more often by crosses within the same species involving unreduced gametes. In contrast, allopolyploid organisms result from hybridization between different species, followed by chromosome doubling [15]. Somatic doubling of diploid and triploid *H. macrophylla* cultivars was previously achieved through Trifluralin-mediated polyploidization, and tetraploid and hexaploid *H. macrophylla* plants were generated [7]. In the present study, we demonstrated that autopolyploid *H. macrophylla* plants can also be generated sexually following different crossing strategies. These strategies include tetraploid plants as well as plants that produce unreduced gametes. As reviewed by Kreiner et al. [16], the production of unreduced gametes has been found across widely disparate phyla. Most individuals of natural populations produce no or low frequencies of 0.1 to 2.0% of unreduced gametes, whereas a few individuals produce also high frequencies, i.e. more than 10%. In *H. macrophylla*, we made similar observations based on flow cytometric analyses of different offspring populations. We found either no, random or predominant polyploidization. Similar findings were also reported for crosses between the diploid *H. macrophylla* cultivars ‘Trophee’, ‘Princess Juliana’ and ‘Zaunkönig’ [11]. The cross ‘Princess Juliana’ x ‘Trophee’ generated 94% triploids, the reciprocal cross no triploids and the cross ‘Zaunkönig’ x ‘Princess Juliana’ 4% triploids [11]. Hence, the production of no, low or high frequencies of unreduced gametes seems to be widespread in cultivated *H. macrophylla*. Reciprocal crosses suggested that predominant polyploidization is depending on the pollen parent. This indicates the production of reduced female gametes and high frequencies of unreduced male gametes in these particular cultivars, namely ‘Hörnli’, ‘Dark Angel’ and ‘Trophee’. However, the source for random polyploidization is unknown and might involve unreduced male and female gametes.

The molecular mechanism of unreduced gamete formation in *H. macrophylla* is completely unknown. As mentioned above, the production of high frequencies of unreduced gametes is cultivar-specific, and therefore genetically determined. Shared genetics between ‘Trophee’, ‘Hörnli’ and ‘Dark Angel’ seems to be unlikely. ‘Trophee’ was bred by the French breeder Lemoine in 1919, whereas ‘Hörnli’ was breed 1952 in Wädenswil, Switzerland, and ‘Dark Angel’ by the German breeder Engel in 2004. Thus, the phenomenon of unreduced pollen formation might have arisen several times independently or is more widespread in at least cultivated *H. macrophylla*.

Crosses with ‘Trophee’, ‘Hörnli’ and ‘Dark Angel’ as pollen parent resulted in different percentages of polyploidized offspring. Crosses with ‘Dark Angel’ as pollinizer yielded 50, 68 or 75% polyploid offspring in combination with different seed partners, whereas ‘Hörnli’ and ‘Trophee’ as pollinizers yielded 94% (Fig. 2 and [11]). The reason for partial generation of diploid and polyploidized offspring is unknown. Self-fertilization of the seed partner might produce diploids. Although self-fertilization is unlikely due to the widespread self-sterility in *H. macrophylla*, we had excluded the risk of selfings by performing hand crosses with removal of the anthers of closed floral buds before pollination. Hence, ‘Trophee’, ‘Hörnli’ and ‘Dark Angel’ probably produce reduced as well as unreduced pollen simultaneously, resulting in diploid and triploid offspring. In order to determine whether the ratio of diploid and triploid offspring is affected by the pollen parent, the individual cross combination or by environmental parameters needs further investigations such as repeated crosses.

Besides diploid and triploid cultivars, we identified the first tetraploid *H. macrophylla* cultivar. The tetraploid cultivar ‘Benelux’ was bred in 1950 by D. Baardse [17]. This tetraploid cultivar might have developed by fusion of two unreduced gametes. We found 3 out of 6 cross combinations between diploids that produced single offspring plants with 2C DNA contents corresponding to the tetraploid level (Figure S2). Furthermore, the crosses ‘Zorro’ x ‘Choco Bleu’ and ‘Benelux’ x ‘Bela’ showed that plants with tetraploid DNA level can also arise from crosses between triploid and diploid or tetraploid partners, although biased numbers of homologous chromosomes cannot be excluded in this offspring despite its clear tetraploid DNA content. A third way for the generation of tetraploids is the fusion of unreduced gametes of triploids with reduced gametes of diploids as found in other species [18], although we found no evidence for this possibility in our study. Hence, the tetraploid cultivar ‘Benelux’ might be the result of one of these seldom events with unwitting selection by the breeder.

In addition, we also found an aneuploid variety, ‘Mücke II’ [9], in our cultivar collection. This variety looked as normal as euploid cultivars. The origin of this aneuploid variety is unknown. Possibly, this variety originated from an interploid cross including a triploid parent and was selected unwittingly by the breeder. In contrast to many other plant species, triploid and even aneuploid *H. macrophylla* plants are fertile. No significant difference in pollen viability was observed between diploid and polyploid *H. macrophylla* individuals, although the pollen viability differed largely between genotypes. Similar findings were previously reported also for other diploid and triploid *H. macrophylla* cultivars. Jones et al. [8] determined pollen viabilities between 7 and 98%, on average 70%, for 42 diploid cultivars and between 25 and 85%, on average 63%, for 19 triploid cultivars. Alexander [19] found a range between 44 and 87% for 3 diploid and between 48 and 75% for 3 triploid cultivars, on average 65 and 61% for diploid and triploid cultivars. Viable offspring was obtained when triploids were used as seed parent or as pollen parent [8, 19], indicating the production of viable male as well as female gametes in triploids.

Using triploid cultivars as parents, we produced a large number of aneuploid plants in this study. These aneuploids differed strongly in chromosome numbers according to their 2C DNA contents. This finding indicates that triploid individuals of *H. macrophylla* successfully perform meiosis, delivering one or two or - even more disturbed - no or three homologous chromosomes to a gamete as has been observed for example in maize [20]. Astonishingly, these aneuploid gametes were obviously viable and able to fertilize, resulting in the numerous aneuploid plants derived from different crosses. The aneuploid plants also were viable, grew for several years in the greenhouse and - to some extend - produced also seeds in crosses with diploid plants. It is still unknown, how these aneuploid plants balance the effect of biased chromosome numbers.

Most of the aneuploid plants showed an abnormal growth and were unattractive. From a breeder’s point of view, crosses based on triploids and aneuploids are of limited use for cultivar selection. However, a few of these plants developed nearly normal and exhibited new, attractive characteristics of leaves or flowers, color and growth habit. Thus, interploid crosses can be a short-term source for the creation of novelty. However, for complex breeding programs that comprise several generations, e.g. breeding for resistance or stress tolerance, the use and development of euploid plants with homogenous development seems more promising.

Moreover, the discovery of polyploid *H. macrophylla* plants and the knowledge about interploid crossing behaviors might support breeding programs regarding interspecific crosses. Interspecific crosses in the genus *Hydrangea* are highly interesting for breeders who aim to combine e.g. winter hardiness and brightly colored flowers. The genus *Hydrangea* includes more than 208 species varying in chromosome number (*2n* = *2x* = 30, 34, 36 or 38 chromosomes) und 2C DNA content (1.95 to 5.00 pg). Most of these species are diploid, but some contain also triploid, tetraploid and/or hexaploid individuals [6, 21, 22]. Interspecific crosses using *H. macrophylla* as one parent succeeded already in combination with *H. angustipetala* and *Dichroa febrifuga* [23, 24]. In contrast, crosses between *H. macrophylla* and *H. paniculata*, *H. quercifolia* or *H. arborescens* failed widely [25–27]. These failures might be caused by choosing unwittingly triploid individuals as parents [27] yielding in aneuploid offspring or by genetic or cytogenetic incompatibilities as suggested by Reed et al. [24, 27]. However, crossing a tetraploid *H. macrophylla* plant with a tetraploid individual of another *Hydrangea* species might overcome this incompatibility and might result in allotetraploid hybrids.

## Conclusions

In the present study, we demonstrated that diploid, triploid, tetraploid and even aneuploid *H. macrophylla* are mostly fertile and produce viable offspring in interploid crosses. Neither triploidy nor aneuploidy cause crossing barriers. Furthermore, we showed that targeted breeding of polyploid *H. macrophylla* plants is possible. We proved that triploid and even tetraploid offspring can be generated by crossing diploid individuals with diploids that form unreduced pollen, by crossing diploid with tetraploid individuals or by crossing triploids either with diploid or with tetraploid plants. Based on the flowcytometric data of the resulting F_1_ populations of our study, we suggest that diploid *H. macrophylla* plants produce usually reduced female gametes, but reduced or unreduced male gametes depending on the cultivar. Triploid cultivars obviously form aneuploid male and female gametes with varying numbers of chromosomes, while tetraploid individuals generate reduced *1n* = *2x* female and male gametes.

Based on these findings, we conclude that targeted breeding of triploid *H. macrophylla* plants can be realized according to the above-mentioned crossing strategies. Furthermore, we assume that also controlled breeding of tetraploid *H. macrophylla* plants is possible by crossing either tetraploid with diploid individuals that develop unreduced pollen or later on by realizing crosses between tetraploids. Thus, targeted breeding of polyploid *H. macrophylla* offspring involving diploids with formation of unreduced pollen and tetraploid plants is possible. Tetraploid plants might be useful in interspecific crosses within the genus *Hydrangea*.

## Methods

### Plant material

In this study, we used 19 commercially available cultivars of *H. macrophylla* (Table 1) for crossing experiments. Plants of these cultivars were provided by the company Kötterheinrich-Hortensienkulturen, Lengerich, Germany. The plants were kept in 17 cm pots filled with Einheitserde® CL Hortensien blau and cultivated in frost-free greenhouses in Erfurt, Germany or in frost-free greenhouses of Kötterheinrich-Hortensienkulturen in Lengerich, Germany. Plants were fertilized with Universol® blue 0.1% (Everris International BV) and irrigated as necessary. Each year in June or July, all plants were pruned. Plants in full bloom were used for crossings and pollen analyses.

### Quantification of pollen viability

For each cultivar, 3 pollen mixes from non-decorative flowers were stained with fluorescein diacetate (FDA) solution as described by Behrend et al. [28]. Eighty-eight to 375 pollen clusters were analyzed per pollen mix. Green-fluorescent pollen kernels were recorded as viable. The pollen viability of diploids and triploids was statistically analyzed using two-tailed Student’s t test with α = 0.05.

### Crossings and seed harvest

Mature inflorescences were selected for crosses. All open flowers were removed. Per cross, 20 to 40 floral buds of an inflorescence were used for hand crossings. Hand crossings were performed by removing the anthers of closed floral buds and putting pollen of the desired crossing partner on the stigmas. Finally, these inflorescences were covered with Crispac-bags (Baumann Saatzuchtbedarf, Waldenburg, Germany) to prevent open-pollination. Selfings were realized by covering inflorescences before anthesis with Crispac-bags (Baumann Saatzuchtbedarf, Waldenburg, Germany).

Seeds were harvested in December by cutting the inflorescences. Dry seed capsules were manually opened. Seeds were collected, counted and stored in small glass tubes in the dark at room temperature until sowing. A cross was defined as successful when more than 20 seeds were obtained and as difficult when only 1–20 seeds were obtained. Crosses that yielded no seeds were classified as not successful.

### Cultivation of F_1_ progenies

Seeds were sown in 250 ml plastic boxes filled with Einheitserde® SP VM. After watering, the boxes were closed with plastic lids. Germination occurred in a climate chamber under a day-night rhythm of 16 h light with 50–70 μmol m^− 2^ s^− 1^ light intensity and 8 h darkness at constant 23 °C. Germination rates were determined 6 to 8 weeks after sowing. After germination, the lids were removed and the boxes were transferred in a greenhouse with 16 h light and 20 °C during the day and 18 °C during the night. When the seedlings had developed 2 to 4 leaves, they were transferred in 8 × 13 multiwell trays filled with Einheitserde® CL P. Depending on the plant size, plants were transferred to 12 and 17 cm pots filled with Einheitserde® CL Hortensien blau and cultivated in a frost-free greenhouse without additional light supply. Plants were fertilized with Universol® blue 0.1% (Everris International BV) and irrigated as necessary.

Germination rates of selected crosses were determined based on 33 to 540 sown seeds.

### Flow cytometry

Flow cytometry was performed on a Partec CyFlow Space analyzer with a 488 nm blue solid state laser. *Pisum sativum* L. ‘Ctirad’ (2C = 9.09 pg) and *Secale cereale* L. ‘Daňkovské’ (2C = 17.05 pg according to *P. sativum* L. ‘Ctirad’) were used as internal standards. Leaf samples of *H. macrophylla* and internal standard were homogenized in Galbraith’s buffer (45 mM MgCl_2_, 20 mM MOPS, 30 mM sodium citrate, 0.1% (v/v) Triton X-100, pH 7, freshly supplemented with 50 μg/ml propidium iodide, 50 μg/ml RNase A and 1% (w/v) PVP 25) according to Doležel et al. [29]. The homogenate was passed through a 30 μm CellTrics filter (Partec) and analyzed at the Partec CyFlow Space analyzer at a flow rate of 0.1 μl/s. For each leaf sample-standard-mixture, about 10,000 nuclei were analyzed, yielding in about 5000 nuclei per sample. Data analysis was performed using the software FloMax version 2.70 (Quantum Analysis GmbH). High quality peaks were determined at CV < 5%. The 2C DNA content of each sample was calculated as follows: 2C_sample_ = mean fluorescence value of sample * 2C DNA content of the corresponding internal standard [pg] / mean fluorescence value of the corresponding internal standard. We analyzed 1 to 9 samples per parental plant and one sample per offspring plant.

### Chromosome counting

Chromosomes of macerated root tips were prepared and stained with DAPI as described in detail by Hempel et al. [9]. Chromosome counts were made from at least 5 metaphase cells per cultivar.

## Supplementary Information

**Additional file 1: Table S1.** Test crosses of putative diploid, triploid and aneuploid F_1_ plants derived from interploid crosses with diploid *H. macrophylla* cultivars ‘Bläuling’, ‘Libelle’, ‘Baby Blue’ and ‘Sheila’, respectively.

**Additional file 2: Figure S1.** Exemplary chromosome metaphases of diploid, triploid, tetraploid and aneuploid *H. macrophylla* plants. **Figure S2.** 2C DNA contents of F_1_ plants from different intraploid and interploid cross combinations.

## Data Availability

The datasets used and/or analyzed in this study are available from the corresponding author on reasonable request.
