# Supplementary material for: Targeted generation of polyploids in Hydrangea macrophylla through cross-based breeding
Source: BMC Genet. 2020 Dec 7;21:147. doi: 10.1186/s12863-020-00954-z (PMC7720383; doi:10.1186/s12863-020-00954-z)
Supplement: Supplementary file 1 — Additional file 1: Table S1. Test crosses of putative diploid, triploid and aneuploid F1 plants derived from interploid crosses with diploid H. macrophylla cultivars ‘Bläuling’, ‘Libelle’, ‘Baby Blue’ and ‘Sheila’, respectively. [file 12863_2020_954_MOESM1_ESM.docx]

## Supplementary

Table S1 Test crosses of putative diploid, triploid and aneuploid F_1_ plants derived from interploid crosses with diploid *H. macrophylla* cultivars ‘Bläuling’, ‘Libelle’, ‘Baby Blue’ and ‘Sheila’, respectively. Crossing success and germination rate (%) were determined. Crosses with more than 20 seeds were defined as successful, with 1-20 seeds as difficult and without seeds as not successful.

| F_1_ plant  (2C DNA content) | Derived from | Pollen viability  [%] | Crossing partner | | | |
| --- | --- | --- | --- | --- | --- | --- |
|  |  |  | ‘Bläuling’ | ‘Libelle’ | ‘Baby Blue’ | ‘Sheila’ |
| E15_022_001  (7.72 pg) | Sweet Dreams x Blaumeise  *2x* x *3x* | n.d. | - | - | 1) successful (66%)  2) successful (68%) | 1) successful (65%)  2) successful (71%) |
| E15_022_003  (6.17 pg) |  | no pollen produced | - | - | 1) difficult  - | 1) not successful  - |
| E15_022_004  (4.38 pg) |  | 29.2 ± 9.6 | - | - | 1) successful (76%)  2) successful (71%) | 1) successful (23%)  2) successful (56%) |
| E15_022_010  (4.45 pg) |  | 9.4 ± 0.7 | - | - | 1) successful (58%)  2) successful (88%) | -  2) successful (64%) |
| E15_005_002  (4.61 pg) | Blaumeise x Sweet Dreams  *3x* x *2x* | 3.8 ± 0.6 | 1) not successful  - | - | 1) not successful  2) not successful | - |
| E15_005_006  (4.98 pg) |  | no pollen produced | 1) not successful  - | - | 1) not successful  - | - |
| E15_005_010  (4.75 pg) |  | 24.0 ± 1.3 | -  2) successful (66%) | - | -  2) successful (90%) | - |
| E15_005_011  (5.12 pg) |  | no pollen produced | 1) not successful  - | - | 1) not successful  - | - |
| E15_005_017  (4.62 pg) |  | 42.5 ± 10.1 | 1) difficult  2) successful (87%) | - | 1) difficult  2) successful (65%) | - |
| E15_005_019  (5.11 pg) |  | no pollen produced | 1) not successful  - | - | 1) not successful  - | - |
| E15_023_001  (6.57 pg) | Zorro x Choco Bleu  *3x* x *2x* | 14.6 ± 1.5 | - | - | 1) successful (49%)  2) not successful | 1) successful (50%)  2) difficult |
| E15_023_002  (6.52 pg) |  | 6.7 ± 1.3 | - | - | 1) successful (44%)  2) not successful | 1) not successful  2) difficult |
| K13_541_009  (4.49 pg) |  | no pollen produced | - | - | 1) not successful  - | 1) not successful  - |
| K13_541_067  (4.58 pg) |  | 24.9 ± 6.1 | - | - | 1) not successful  - | 1) difficult  - |
| K14_540_003  (6.65 pg) | Benelux x Forever Pink  *4x* x *2x* | 14.3 ± 7.7 | 1) successful (31%)  2) successful (45%) | - | - | - |
| K14_540_008  (6.71 pg) |  | 18.2 ± 4.0 | 1) difficult  2) difficult | - | - | - |
| K14_540_017  (7.04 pg) |  | 15.0 ± 6.1 | 1) successful (51%)  2) difficult | - | - | - |
| K14_540_021  (6.46 pg) |  | 17.9 ± 5.6 | 1) difficult  2) successful (35%) | 1) not successful  - | - | - |
| K14_540_113  (6.98 pg) |  | 24.2 ± 6.1 | 1) difficult  2) successful (60%) | - | - | - |
| E15_001_006  (6.77 pg) | Bela x Benelux  *3x* x *4x* | 12.3 ± 2.9 | 1) successful (40%)  - | 1) successful (43%)  - | - | - |
| E15_003_008  (7.10 pg) |  | 16.6 ± 7.2 | 1) successful (37%)  2) difficult | 1) successful (32%)  - | - | - |
| E15_003_018  (7.58 pg) |  | no pollen produced | 1) successful (26%)  2) difficult | 1) successful (100%)  - | - | - |
| E15_003_025  (6.65 pg) |  | 9.1 ± 5.0 | 1) successful (42%)  2) difficult | 1) successful (21%)  - | - | - |

Reciprocal crosses: 1) F_1_ plant x diploid *H. macrophylla* cultivar, 2) diploid *H. macrophylla* cultivar x F_1_ plant, n.d. not determined, - crosses not performed
