# Supplementary material for: Targeted generation of polyploids in Hydrangea macrophylla through cross-based breeding
Source: BMC Genet. 2020 Dec 7;21:147. doi: 10.1186/s12863-020-00954-z (PMC7720383; doi:10.1186/s12863-020-00954-z)
Supplement: Supplementary file 2 — Additional file 2: Figure S1. Exemplary chromosome metaphases of diploid, triploid, tetraploid and aneuploid H. macrophylla plants. Figure S2. 2C DNA contents of F1 plants from different intraploid and interploid cross combinations. [file 12863_2020_954_MOESM2_ESM.pdf]

Supplementary

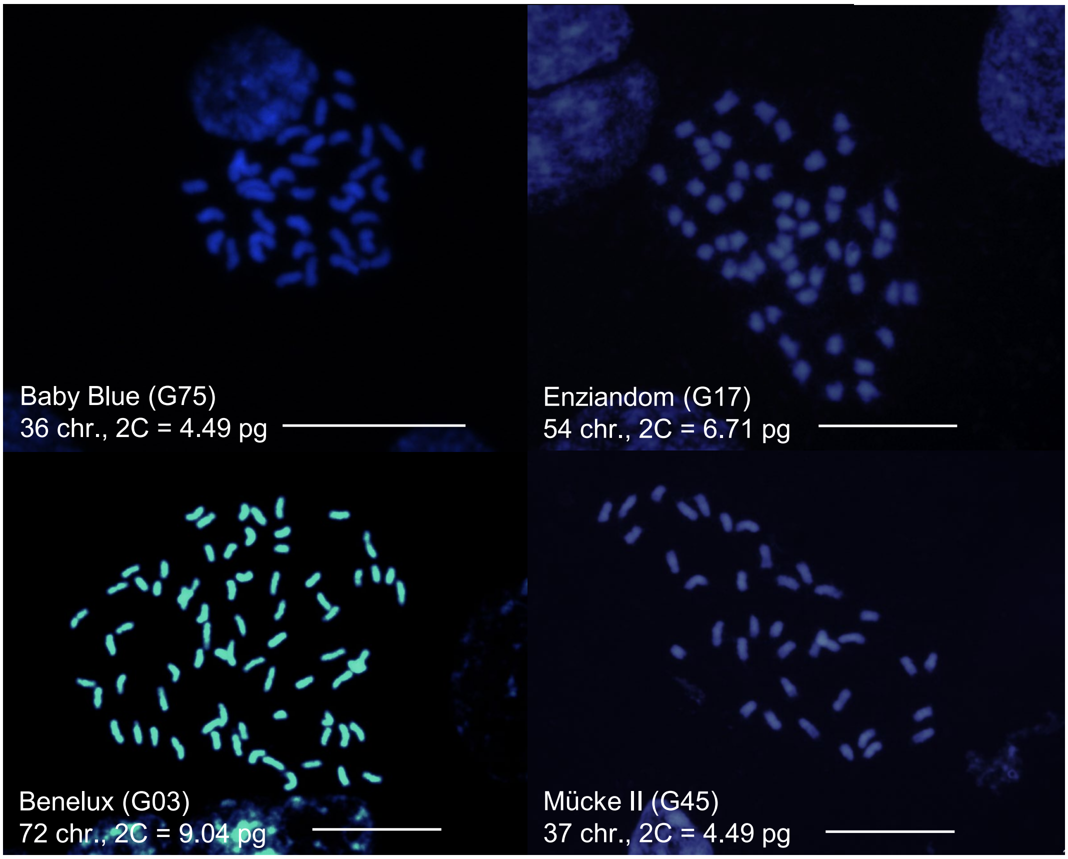

Figure S1. Exemplary chromosome metaphases of diploid, triploid, tetraploid and aneuploid *H. macrophylla* plants. The cultivar names and corresponding SSR fingerprint IDs refer to Hempel et al. [9] and Tränkner et al. [12]. Bar 20 µm.

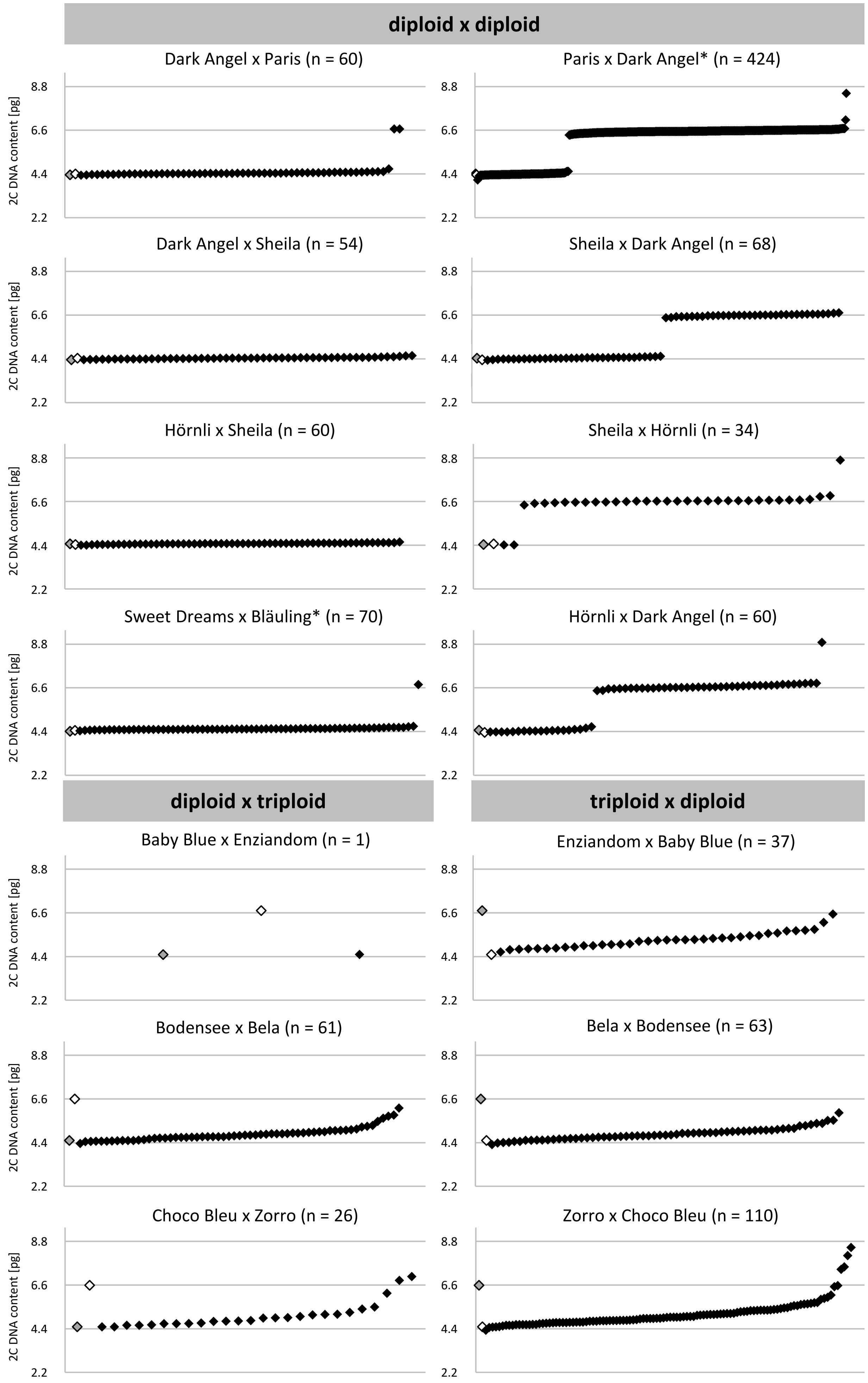
diploid x diploid
Dark Angel x Paris (n = 60)
Paris x Dark Angel* (n = 424)
Dark Angel x Sheila (n = 54)
Sheila x Dark Angel (n = 68)
Hörnli x Sheila (n = 60)
Sheila x Hörnli (n = 34)
Sweet Dreams x Bläuling* (n = 70)
Hörnli x Dark Angel (n = 60)
diploid x triploid
triploid x diploid
Baby Blue x Enziandom (n = 1)
Enziandom x Baby Blue (n = 37)
Bodensee x Bela (n = 61)
Bela x Bodensee (n = 63)
Choco Bleu x Zorro (n = 26)
Zorro x Choco Bleu (n = 110)
2C DNA content [pg]
8.8
6.6
4.4
2.2

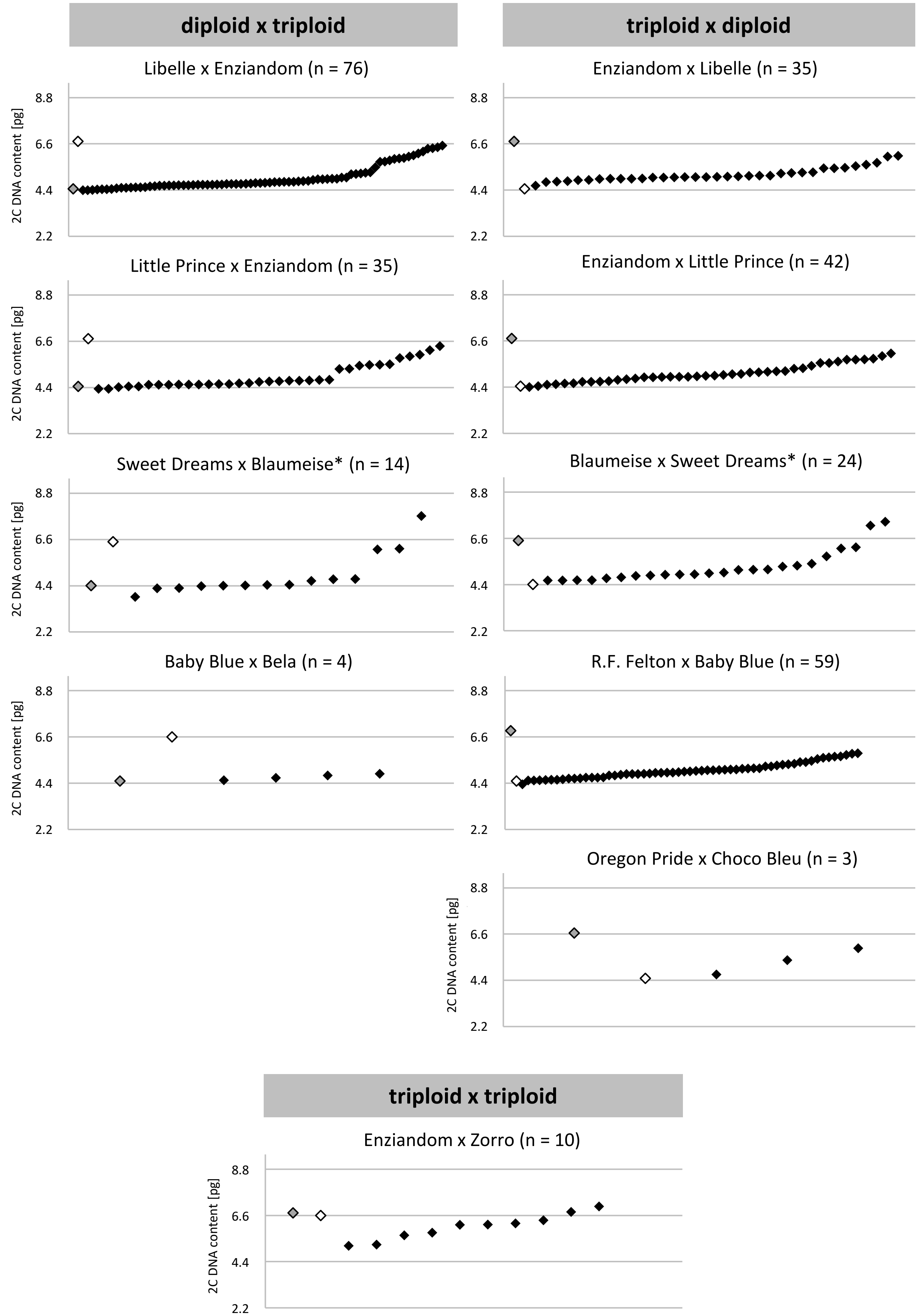
diploid x triploid
triploid x diploid
Libelle x Enziandom (n = 76)
Enziandom x Libelle (n = 35)
Little Prince x Enziandom (n = 35)
Enziandom x Little Prince (n = 42)
Sweet Dreams x Blaumeise* (n = 14)
Blaumeise x Sweet Dreams* (n = 24)
Baby Blue x Bela (n = 4)
R.F. Felton x Baby Blue (n = 59)
Oregon Pride x Choco Bleu (n = 3)
triploid x triploid
Enziandom x Zorro (n = 10)
2C DNA content [pg]
8.8
6.6
4.4
2.2

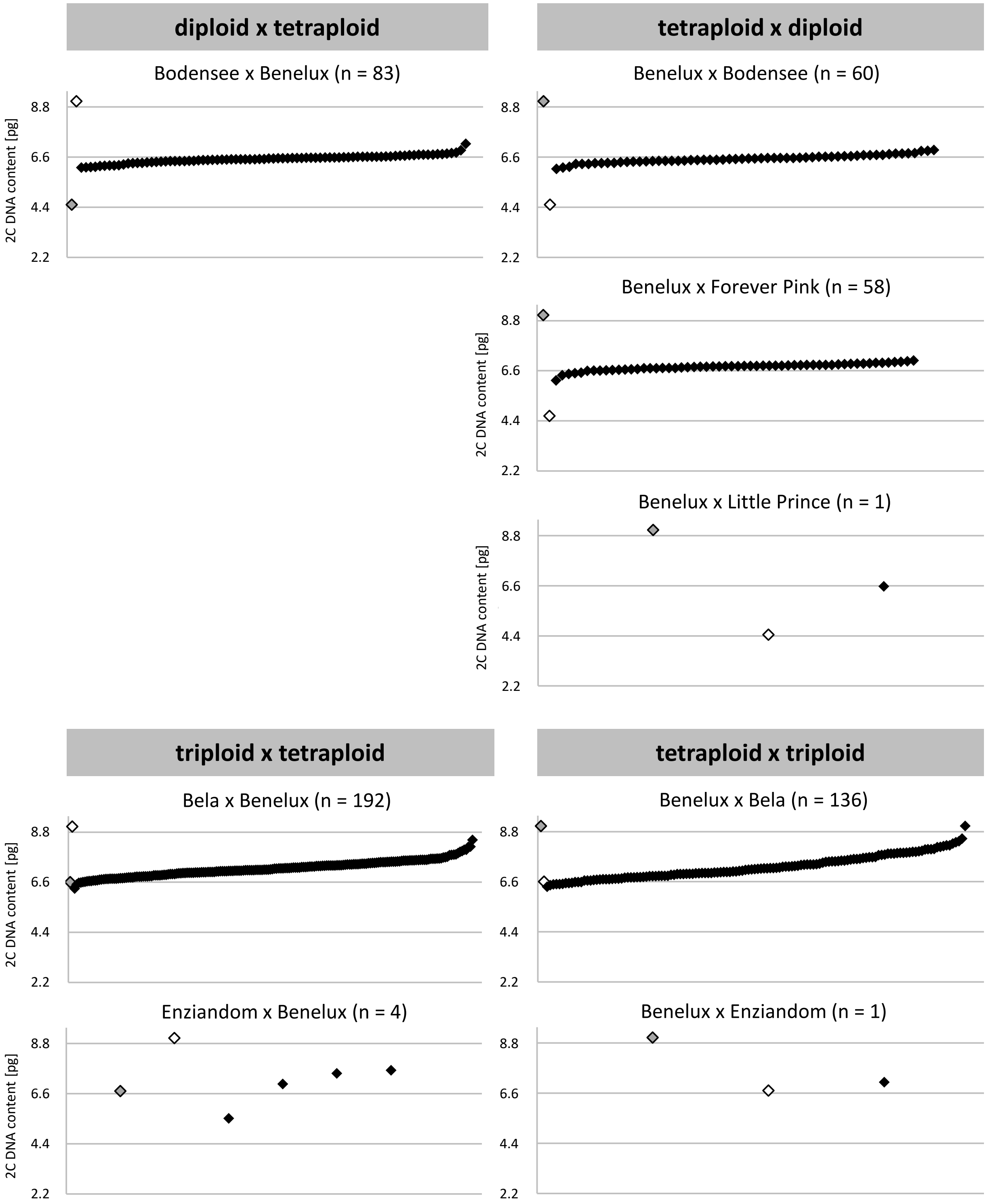

Figure S2. 2C DNA contents of F_1 plants from different intraploid and interploid cross combinations. The 2C DNA contents of F_1 plants are shown in black, the 2C DNA contents of the corresponding maternal and paternal parents are shown in grey and white, respectively. n gives the number of analyzed F_1 plants. *data already presented in Tränkner et al. [12, 14].
